# Olmesartan Potentiates the Anti-Angiogenic Effect of Sorafenib in Mice Bearing Ehrlich's Ascites Carcinoma: Role of Angiotensin (1–7)

**DOI:** 10.1371/journal.pone.0085891

**Published:** 2014-01-22

**Authors:** Mohammad M. Abd-Alhaseeb, Sawsan A. Zaitone, Soad H. Abou-El-Ela, Yasser M. Moustafa

**Affiliations:** 1 Department of Pharmacology and Toxicology, Faculty of Pharmacy and Pharmaceutical Industries, Sinai University, Arish, Egypt; 2 Department of Pharmacology and Toxicology, Faculty of Pharmacy, Suez Canal University, Ismailia, Egypt; 3 Department of Biochemistry, Faculty of Pharmacy and Pharmaceutical Industries, Sinai University, Arish, Egypt; University of South Alabama, United States of America

## Abstract

Local renin-angiotensin systems exist in various malignant tumor tissues; this suggests that the main effector peptide, angiotensin II, could act as a key factor in tumor growth. The underlying mechanisms for the anti-angiogenic effect of angiotensin II type 1 receptor blockers need to be further evaluated. The present study was carried out to investigate the anti-angiogenic effect of olmesartan alone or in combination with sorafenib, an angiotensin (1–7) agonist or an angiotensin (1–7) antagonist in Ehrlich's ascites carcinoma-bearing mice. The tumor was induced by intradermal injection of Ehrlich's ascites carcinoma cells into mice. Tumor discs were used to evaluate the microvessel density; the serum levels of vascular endothelial growth factor (VEGF) and serum insulin-like growth factor I (IGF-I); and their intratumoral receptors, VEGF receptor-2 and IGF-I receptor, respectively. All parameters were determined following the treatment course, which lasted for 21 days post-inoculation. Monotherapy with olmesartan and its combination with sorafenib resulted in a significant reduction in microvessel density and serum levels of VEGF and IGF-I, as well as their intratumoral receptors. In addition, the combination of olmesartan (30 mg/kg) with an angiotensin (1–7) agonist reduced the microvessel density, IGF-I serum levels and the levels of its intratumoral receptor. In conclusion, olmesartan reduced the levels of the angiogenesis markers IGF-I and VEGF and down-regulated the intratumoral expression of their receptors in a dose-dependent manner, and these effects were dependent on the angiotensin (1–7) receptor. These results suggest that olmesartan is a promising adjuvant to sorafenib in the treatment of cancer.

## Introduction

Angiogenesis is the process by which new capillaries grow from existing blood vessels[1]. To develop, tumors require the formation of fresh blood vessels from pre-existing ones[2]. In many tumor systems, IGF-I, IGF-II and the insulin-like growth factor receptor-I are over expressed [3]. IGF-I plays a role in the induction of cell proliferation and tumor angiogenesis, and these effects have also been attributed to the induction of VEGF [3]. Vascular endothelial growth factors are a group of cytokines that are involved in essential physiological processes and are aberrantly expressed in many pathologies. VEGF binds to a tyrosine kinase receptor known as VEGF receptor-2[4].

The renin-angiotensin system plays an important role in controlling blood pressure, cardiovascular and renal function and cell growth [5].The renin-angiotensin system is a hormone system that is activated when renin is released, resulting in the cleavage of angiotensinogen into angiotensin I. Angiotensin I is then converted into angiotensin II and angiotensin (1–7) by angiotensin-converting enzymes [6]. Local renin-angiotensin systems exist in various malignant tumor tissues, which suggests that the main effector peptide, angiotensin II, could act as a key factor in tumor growth and angiogenesis via the angiotensin II type 1 receptor [7].

During the progression from normal to malignant phenotypes, the angiotensin II type 1 receptor is often up-regulated, which suggests a correlation between the renin-angiotensin system and tumor progression [8]. Angiotensin II activates neovascularization via the induction of VEGF release [9]. The Mas1 oncogene (MasR) represents another rennin-angiotensin system receptor that binds angiotensin (1–7) peptide[10]. Angiotensin (1–7) can be produced from AngI or AngII via endo- or carboxy-peptidases respectively [11] and has apoptotic and anti-proliferative actions[12]. In addition, angiotensin (1–7) inhibits the growth of vascular smooth muscles both *in vitro* and *in vivo*, and this effect is blocked by the angiotensin (1–7) receptor antagonist [D-Ala^7^]-Ang-(1–7) (A-779 peptide) [13].

Sorafenib, a multi-kinase inhibitor taken orally, has been shown to suppress tumor growth by inhibiting serine/threonine kinases, such as c-RAF, platelet-derived growth factor receptor, fms-like tyrosine kinase 3, Ret, proto-oncogene c-Kit and the receptor tyrosine kinases VEGF receptors 2 and 3 [14]. Sorafenib has been used to treat renal cell carcinoma [15], but it results in an increased risk of hypertension[16], bleeding, skin reactions on the hands and feet and arterial thromboembolism [16], [17].

Ehrlich's ascites carcinoma (EAC) is an undifferentiated carcinoma [18]. It has high transplantable capability, no-regression, rapid proliferation, short life span, 100% malignancy and does not have tumor-specific transplantation antigen (TSTA)[19]. EAC resembles human tumors and is used in many studies as experimental model to study the antitumor or anti-angiogenic activities of drugs or natural compounds [18], [20], [21].

The objective of the current study was to further elucidate the anti-angiogenic effect of olmesartan, an angiotensin II type 1 receptor blocker, and to examine the impact of concurrent administration of an angiotensin (1–7) agonist or an angiotensin (1–7) antagonist on the anti-angiogenic effect of olmesartan. Additionally, the study sought to test whether olmesartan could potentiate the anti-angiogenic effect of sorafenib in mice bearing Ehrlich's ascites carcinoma. This aim was achieved by determining the serum levels of IGF-I and VEGF, as well as the intratumoral expression of their receptors.

## Materials and Methods

### Animals

Female Swiss albino mice, weighing 20–30 g, were purchased from the Modern Veterinary Office for Laboratory Animals (Cairo, Egypt). Mice were housed in polyethylene cages under controlled laboratory conditions (25±1°C temperature, constant relative humidity and normal dark/light cycle). Food and water were provided *ad libitum*. All experimental protocols were approved by The Animal Care and Use Committee at the Faculty of Pharmacy, Suez Canal University.

### Drugs and chemicals

Olmesartan medoxomil was purchased from Daiichi Sankyo Pharmaceutical Co. (Tokyo, Japan) and was dissolved in dimethylsulfoxide (DMSO; Sigma-Aldrich®, MO, USA). Sorafenib tosylate was purchased from Bayer AG (Leverkusen, Germany). (D-Ala^7^)-angiotensin I/II (1–7) trifluroacetate salt (A-779 Peptide, angiotensin [1]–[7] antagonist) and angiotensin I/II (1–7) trifluroacetate salt (Angiotensin [1]–[7] agonist) were purchased from Bachem AG (Bubendorf, Zurich). Rabbit polyclonal antibodies against mouse VEGF receptor type-2 were purchased from Bio SB (Santa Barbara, California, USA). Monoclonal antibodies against mouse CD_31_ and IGF-I receptors were purchased from Thermo Fisher Scientific® (Fremont, USA). 3, 3′-diaminobenzidine (DAB) was purchased from Sigma-Aldrich® (MO, USA). All other chemicals were supplied in analytical grades from commercial sources.

### Induction of solid tumors in mice

Ehrlich's ascites carcinoma is used as ascites or as a solid form [21] and easy to grow in suspension in the peritoneal cavity of mice. Further, EAC suspension contains homogeneous free tumor cells so it has a transplantable capacity for certain quantitative tumor cells to another mouse [21], [22]. Finally, EAC cell line is easily prepared, grown and safe model for *in-vivo* experiments [19], [22].

The Ehrlich's ascites carcinoma cell line was purchased from the Tumor Biology Department, National Cancer Institute, Cairo University (Cairo, Egypt). The Ehrlich's ascites carcinoma cells were prepared under aseptic conditions. The viability of the Ehrlich's ascites carcinoma cells was tested using Trypan blue dye exclusion technique [23]. Ehrlich's ascites carcinoma cells were suspended in normal saline; each 0.1 mL of this diluted suspension contained 2.5 million Ehrlich's ascites carcinoma cells. At the first day of the experiment, mice were inoculated intradermally with 0.1 mL of the Ehrlich's ascites carcinoma suspension bilaterally on the lower ventral side.

### Experimental design

Ninety mice were randomly divided into nine groups, ten mice each. Group I: normal mice that were injected with normal saline (0.1 mL/mouse, i.d.) at the first day of the experiment on the lower ventral side and then treated with saline (5 mL/kg/day, p.o.) starting from day 8 until the last day of the experiment. One week after inoculation with the tumor cells (day 8), tumor growth was confirmed and therapeutic regimens were launched as follows. Group II: mice treated with DMSO (5 mL/kg/day, p.o.), and served as the EAC-control group. Group III: mice treated with sorafenib (30 mg/kg/day, p.o.) [24]. Group IV-VI: mice treated with olmesartan (3, 10 or 30 mg/kg/day, p.o.), respectively [25]. Group VII: mice treated with a combination of sorafenib (30 mg/kg/day, p.o.) and olmesartan (30 mg/kg/day, p.o.). Group VIII: mice treated with olmesartan (30 mg/kg/day, p.o.) and the angiotensin (1–7) agonist (30 µg/kg/day, i.p.) [26]. Group IX: mice were treated with olmesartan (30 mg/kg/day, p.o.) and the angiotensin (1–7) antagonist (A-779 peptide) (3.3 mg/kg/trice weekly, i.p.) [27]. In general, olmesartan and sorafenib were administered daily by gastric gavage in a volume of 5 mL/kg.Whereas, the angiotensin (1–7) agonist or the angiotensin (1–7) antagonist were administered intraperitoneally. All treatments were launchedon day 8 and continued for 21 days (a three-week therapeutic period).

### Collection of serum samples and dissection of tumor discs

At the end of the experiment (day 28), blood samples were withdrawn from each mouse from the orbital sinus under light ether anesthesia. Blood samples were allowed to stand for 30 min at room temperature and then, centrifuged at 1000×*g* for 10 min. Serum samples were separated and stored at −20°C until used for ELISA assays. After that, mice were sacrificed by cervical dislocation and tumor discs were dissected, weighed and fixed in 10% phosphate-buffered formalin. All paraffin-embedded tissues were sectioned at 4 µm and prepared for hematoxylin and eosin (H&E) staining and for immunohistochemical staining of CD_31_, IGF-I receptors and VEGF receptors-2.

### Determination of serum IGF-I and VEGF

Serum IGF-I and VEGF levels were determined using enzyme-linked immune sorbent assay (ELISA) kits purchased from Biorbyt Ltd. (Cambridge, England) and Sun Red Biotechnology Company (Shanghai, China), respectively. The color intensity was measured at 450 nm using a microplate reader (Metertech, M960).

### Histopathological examination, immunohistochemistry and image analysis

Sections were fixed in a 65°C oven for 1 h. Then, the slides were placed in a Coplin jar filled with 50 mL Triology (Cell Marque®, CA-USA) working solution, and the jar was securely positioned in an autoclave. The autoclave at 120°C and maintained for 15 min; after which, the pressure was released, and the Coplin jar was removed and the slides were allowed to cool. After that, sections were washed and immersed in TBS to adjust the pH; this wash step was repeated between each step of the immunohistochemical procedures. Excess serum was drained and 2 drops of the rabbit monoclonal CD_31_ primary antibody, 1∶50 in phosphate buffered saline (PBS) (Thermo Scientific®, Fremont, USA), the ready to use mouse monoclonal antibodies for the IGF-I receptors (Thermo Scientific®, Fremont, USA) or the rabbit polyclonal VEGF receptor-2 antibodies (1∶80 with PBS, Bio SB, Santa Barbara, California, USA) were added to each slide.

Then, slides were incubated in a humidity chamber for 1 h. Next, biotinylated secondary antibodies were applied to each slide for 20 min, followed by a 20 min incubation period with the enzyme conjugate. DAB chromogen was prepared and 3 drops were applied to each slide for 2 min. After that, the DAB was rinsed off, and the slides were counter stained with Mayer's hematoxylin. Finally, cover slipping was performed and the slides were examined under a light microscope (Olympus CX21, Japan). The stained slides were examined to identify the areas of high neovascularization. In each section, the ten most vascular areas were chosen. The photomicrographs were examined using the Image J1.45 F image analysis system (National Institute of Health, USA) to determine the optical density of the immunostaining. All histopathological examinations were performed by an experienced pathologist who was blinded to the experimental groups.

### Statistical Analysis

Results were collected, tabulated and expressed as mean ± S.E.M. Data were analyzed using one-way analysis of variance (ANOVA) followed by Bonferroni's *post-hoc* test. All statistical tests were performed using the Statistical Package for Social Sciences, version 19 (SPSS Software, SPSS Inc., Chicago, USA) and the differences were considered significant when *P*<0.05.

## Results

### Tumor weight

**Figure 1:**
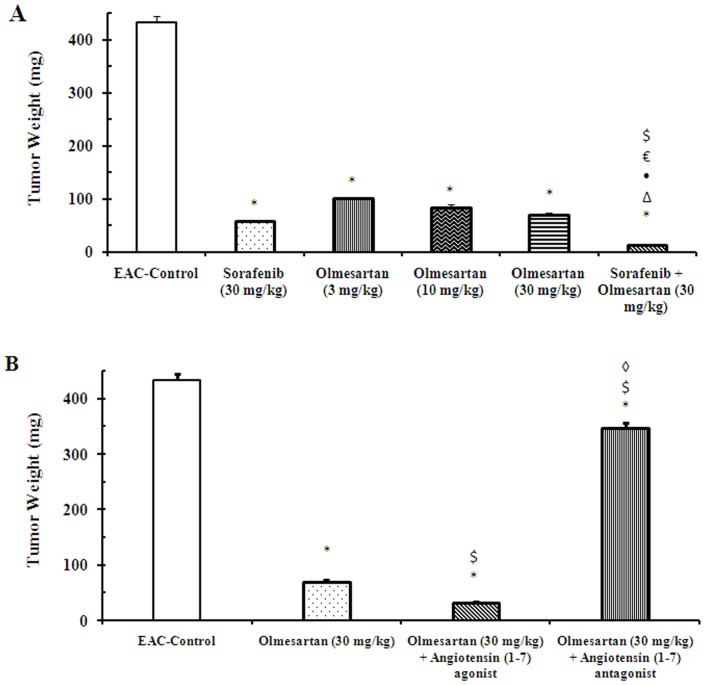
Effect of sorafenib and olmesartan on tumor weight in EAC- bearing mice. **A**) Effect of sorafenib (30 mg/kg), olmesartan (3, 10 or 30 mg/kg) and their combination on tumor weight in EAC-bearing mice. **B**) Effect of concurrent administration of an angiotensin (1–7) agonist (30 µg/kg/day, i.p.) or an angiotensin (1–7) antagonist (3.3 mg/kg/trice/week, i.p.) and olmesartan on the tumor weight of EAC-bearing mice. EAC: Ehrlich's ascites carcinoma. Values are expressed as the mean ± S.E.M. anddata were analyzed using one-way ANOVA followed by Bonferroni's *post-hoc* test at*P*<0.05. *Significantly different from the EAC-control. ^Δ^Significantly different from sorafenib monotherapy. •Significantly different from olmesartan (3 mg/kg) group. ^€^Significantly different from olmesartan (10 mg/kg) group.^$^Significantly different from olmesartan (30 mg/kg) group.^◊^Significantly different from the combination of olmesartan and angiotensin (1–7) agonist.

At the end of the experiment, monotherapy with sorafenib (30 mg/kg), olmesartan (3, 10 or 30 mg/kg) or their combination reduced the tumor weight, compared to the EAC-control mice (Fig. 1A). Concurrent administration of the angiotensin (1–7) agonist with olmesartan (30 mg/kg) reduced the tumor weight, compared to either the EAC-control group or olmesartan (30 mg/kg) group. Meanwhile, concurrent administration of the angiotensin (1–7) antagonist with olmesartan (30 mg/kg) reduced the antitumor effect of olmesartan; the tumor weight in this group was different from the EAC-control group, olmesartan (30 mg/kg) group and angiotensin (1–7) agonist group (Fig. 1B). These results indicated that the anti-angiogenic effect of olmesartan was mediated, at least in part, through the activation of the angiotensin (1–7) receptor.

### Serum level of IGF-I and VEGF

**Figure 2:**
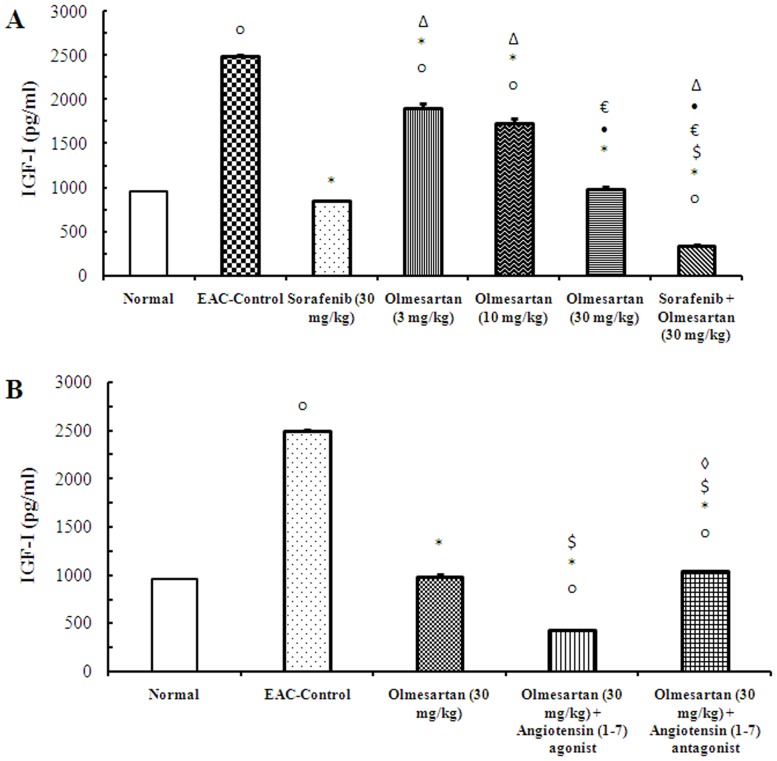
Effect of sorafenib and olmesartan on the serum level of IGF-I in EAC- bearing mice. **A**) Effect of sorafenib (30 mg/kg), olmesartan (3, 10 or 30 mg/kg) and their combination on the serum level of IGF-I in EAC-bearing mice. **B**) Effect of concurrent administration of an angiotensin (1–7) agonist (30 µg/kg/day, i.p.) or an angiotensin (1–7) antagonist (3.3 mg/kg/trice/week, i.p.) and olmesartan on serum level of IGF-I in EAC-bearing mice. EAC: Ehrlich's ascites carcinoma. IGF-1: insulin growth factor-1. Values are expressed as the mean ± S.E.M. and data were analyzed using one-way ANOVA followed by Bonferroni's *post-hoc* test at P<0.05. ^o^Significantly different from the normal group. *Significantly different from EAC-control. ^Δ^Significantly different from sorafenib monotherapy. •Significantly different from olmesartan (3 mg/kg) group. ^€^Significantly different from olmesartan (10 mg/kg) group.^$^Significantly different from olmesartan (30 mg/kg) group.^◊^Significantly different from the combination of olmesartan and angiotensin (1–7) agonist.

The serum level of IGF-I was greater in the EAC-control mice compared to the normal mice. Various pharmacological treatments reduced the serum level of IGF-I compared to the EAC-control group. Further, treatment with a combination of olmesartan (30 mg/kg) and sorafenib significantly reduced the serum level of IGF-I compared to each monotherapy (*P*<0.05, Fig. 2A). In contrast, concurrent administration of the angiotensin (1–7) agonist with olmesartan (30 mg/kg) reduced the serum level of IGF-I compared to monotherapy with olmesartan (30 mg/kg) (*P*<0.05, Fig. 2B).

**Figure 3:**
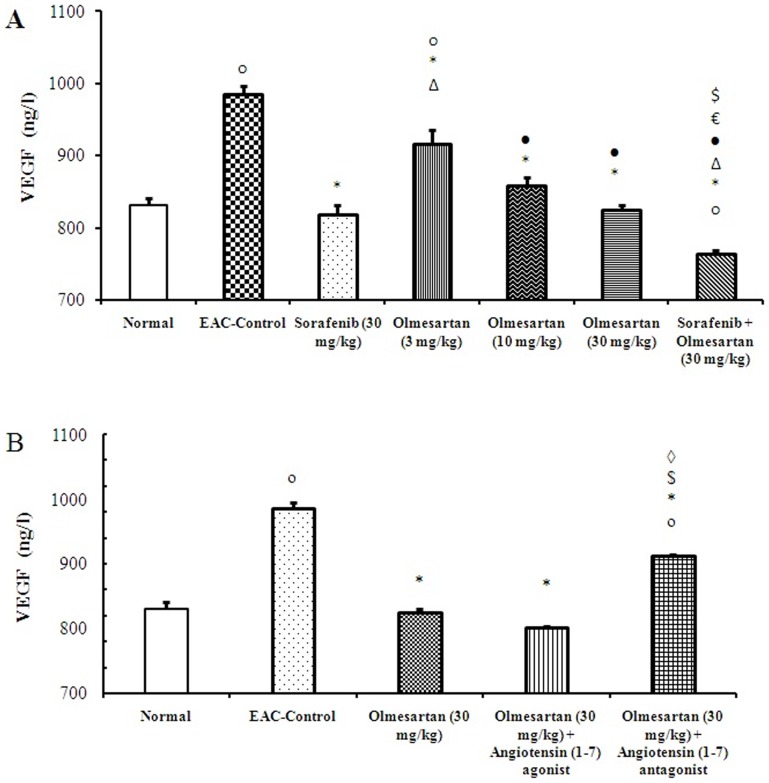
Effect of sorafenib and olmesartan on the serum level of VEGF in EAC- bearing mice. **A**) Effect of sorafenib (30 mg/kg), olmesartan (3, 10 or 30 mg/kg) and their combination on the serum level of VEGF in EAC-bearing mice. **B**) Effect of concurrent administration of angiotensin (1–7) agonist (30 µg/kg/day, i.p.) or angiotensin (1–7) antagonist (3.3 mg/kg/trice/week, i.p.) and olmesartan on the serum level of VEGF in EAC-bearing mice. EAC: Ehrlich's ascites carcinoma. VEGF: vascular endothelial growth factor. Values are expressed as the mean ± S.E.M. and analyzed using one-way ANOVA followed by Bonferroni's *post-hoc* test at *P*<0.05. ^o^Significantly different from the normal group. *Significantly different from EAC-control. ^Δ^Significantly different from sorafenib monotherapy. •Significantly different from olmesartan (3 mg/kg) group.^€^Significantly different from olmesartan (10 mg/kg) group.^$^Significantly different from olmesartan (30 mg/kg) group.^◊^Significantly different from the combination of olmesartan and angiotensin (1–7) agonist.

Comparing the serum level of VEGF on day 28 highlighted a significant increase in the EAC-control group compared to the normal group. Sorafenib or olmesartan (3, 10 or 30 mg/kg), dose dependently, reduced the serum level of VEGF compared to the EAC-control group. In contrast, the combination of olmesartan (30 mg/kg) and sorafenib reduced the serum level of VEGF compared to the corresponding monotherapies (*P*<0.05, Fig. 3A). Concurrent administration of the angiotensin (1–7) agonist with olmesartan (30 mg/kg) reduced the serum level of VEGF compared to the olmesartan monotherapy (30 mg/kg). In contrast, concurrent administration of angiotensin (1–7) antagonist with olmesartan (30 mg/kg) increased the serum level of VEGF compared to the olmesartan (30 mg/kg) group (*P*<0.05, Fig. 3B).

### Optical density of immunostaining for IGF-I receptor and VEGF receptors

**Figure 4:**
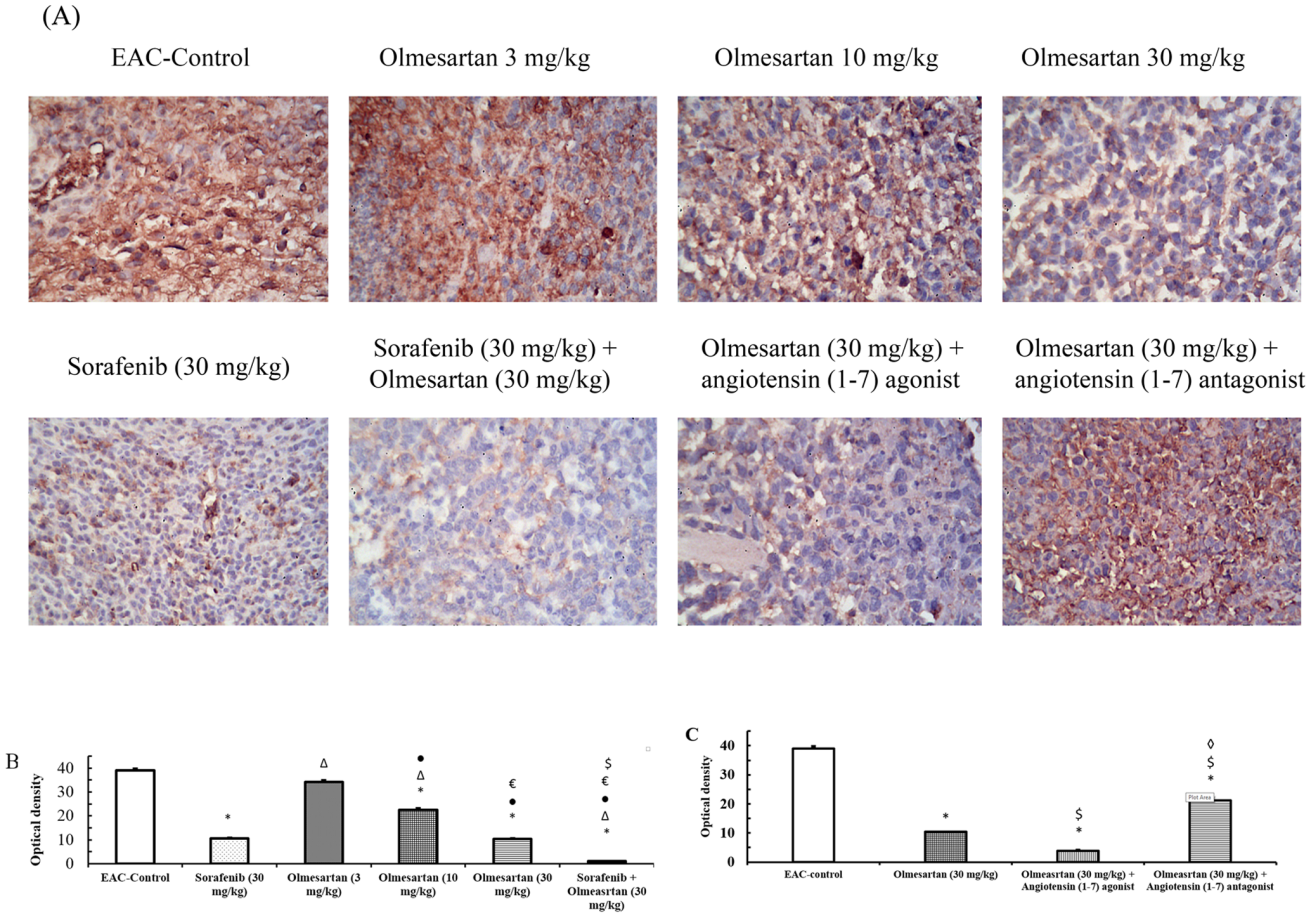
Immunostaining and optical density of IGF receptor type -I. **A**) Immunostaining for IGF receptor type-I in the experimental groups. **B**) Effect of sorafenib (30 mg/kg), olmesartan (3, 10 or 30 mg/kg) and their combination on the optical density of IGF receptor type-I immunostaining. **C**) Effect of concurrent administration of angiotensin (1–7) agonist (30 µg/kg/day, i.p.) or angiotensin (1–7) antagonist (3.3 mg/kg/trice a week, i.p.) and olmesartan on the intratumoral level of IGF receptors type-I. Photomicrographs are captured at 200× magnification. EAC: Ehrlich's ascites carcinoma. Values are expressed as the mean ± S.E.M. and analyzed using one-way ANOVA followed by Bonferroni's *post-hoc* test at *P*<0.05. * Significantly different from EAC-control. ^Δ^Significantly different from sorafenib monotherapy.•Significantly different from olmesartan (3 mg/kg) group. ^€^Significantly different from olmesartan (10 mg/kg) group.^$^Significantly different from the olmesartan (30 mg/kg) group.^◊^Significantly different from the combination of olmesartan and angiotensin (1–7) agonist.

At the end of the experiment, treatment with sorafenib (30 mg/kg) or olmesartan (10 or 30 mg/kg) reduced the optical density for IGF-I receptor immunostaining in the produced solid tumor compared to the EAC-control group. In addition, the combination of olmesartan (30 mg/kg) and sorafenib reduced the optical density for IGF-I receptor immunostaining (*P*<0.05, Fig. 4A and 4B). Concurrent administration of the angiotensin (1–7) agonist with olmesartan (30 mg/kg) reduced the optical density for IGF-I receptor immunostaining compared to olmesartan (30 mg/kg) group. However, concurrent administration of the angiotensin (1–7) antagonist with olmesartan (30 mg/kg) increased the optical density of IGF-I receptor immunostaining compared to olmesartan (30 mg/kg) group (*P*<0.05, Fig. 4A and 4C).

**Figure 5:**
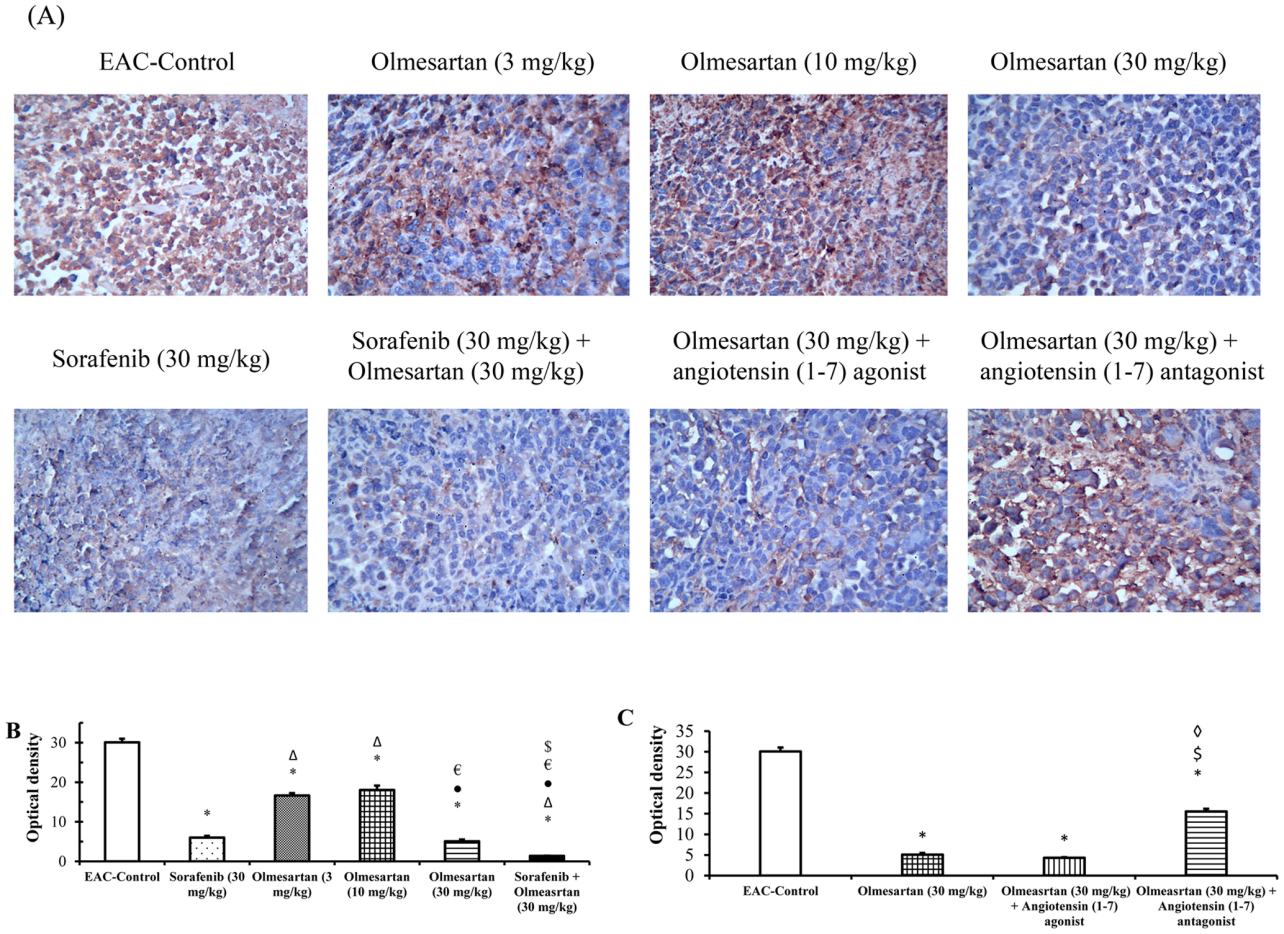
Immunostaining and optical density of VEGF receptor type -2. **A**) Immunostaining for VEGF receptor type-2 in the experimental groups. **B**) Effect of sorafenib (30 mg/kg), olmesartan (3, 10 or 30 mg/kg) and their combination on the optical density of VEGF receptor type-2 immunostaining. **C**) Effect of concurrent administration of angiotensin (1–7) agonist (30 µg/kg/day, i.p.) or angiotensin (1–7) antagonist (3.3 mg/kg/trice/week, i.p.) and olmesartan on the intratumoral levels of VEGF receptors type-2. Photomicrographs are captured at 200× magnification. EAC: Ehrlich's ascites carcinoma. Values are expressed as the mean ± S.E.M. and were analyzed using one-way ANOVA followed by Bonferroni's *post-hoc* at *P*<0.05. *Significantly different from the EAC-control. ^Δ^Significantly different from the sorafenib monotherapy.•Significantly different from olmesartan (3 mg/kg) group. ^€^Significantly different from olmesartan (10 mg/kg) group.^$^Significantly different from olmesartan (30 mg/kg) group.^◊^Significantly different from the combination of olmesartan and angiotensin (1–7) agonist.

Similarly, a difference in the optical density for VEGF receptor type-2 immunostaining was observed among the study groups (*P*<0.05). Olmesartan, dose-dependently, reduced the optical density for these receptors compared to the EAC-control group (Fig. 5A). Combining olmesartan (30 mg/kg) and sorafenib reduced the expression of VEGF receptor type-2 compared to the EAC-control group or compared to the corresponding monotherapies (Fig. 5B).

Further, concurrent administration of the angiotensin (1–7) agonist with olmesartan (30 mg/kg) reduced the optical density for VEGF receptor type-2 immunostaining compared to EAC-control group. In contrast, concurrent administration of the angiotensin (1–7) antagonist with olmesartan (30 mg/kg) increased the expression of VEGF receptor type-2 in comparison to olmesartan (30 mg/kg) group (*P*<0.05, Fig. 5C).

### Effect on intratumoral microvessel density

**Figure 6:**
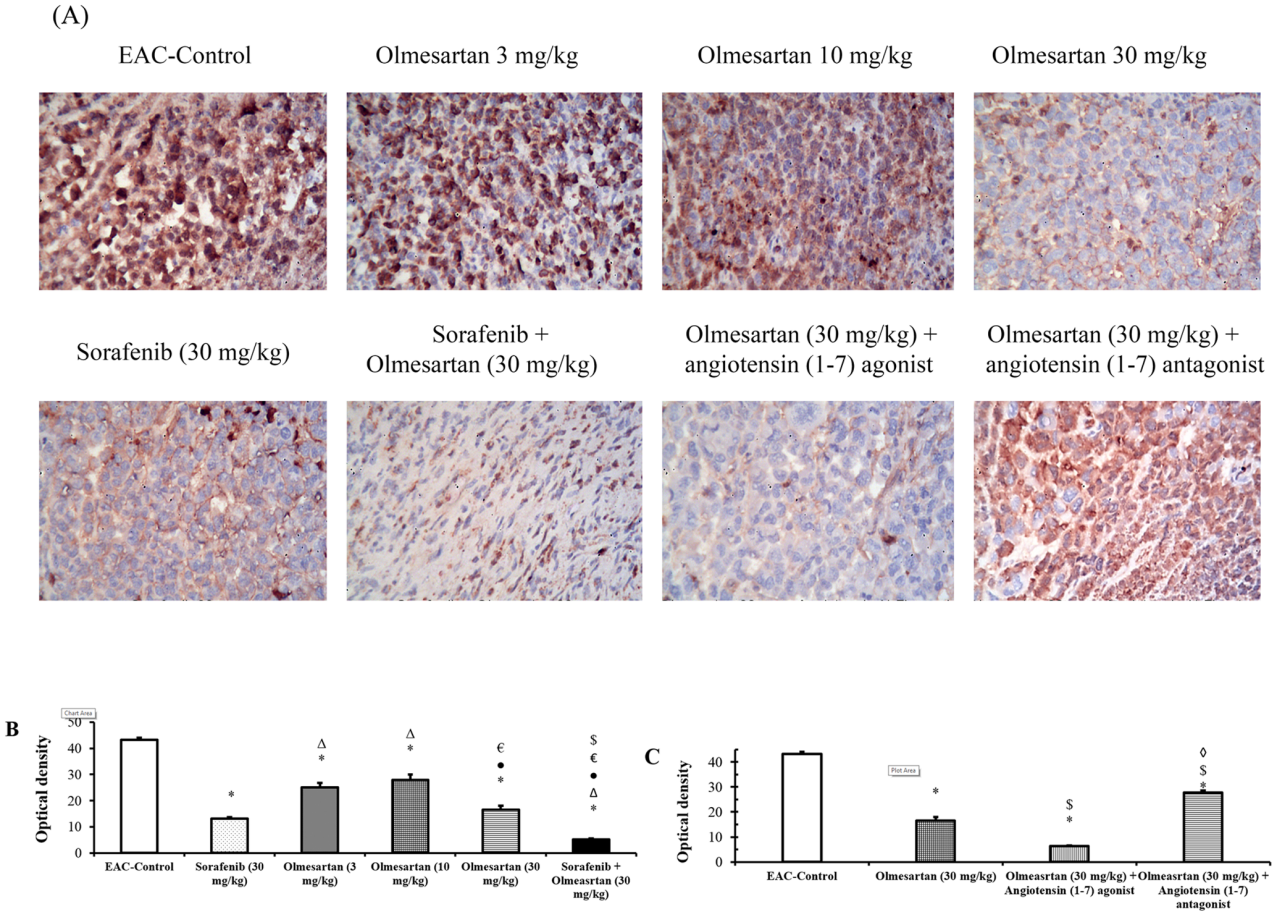
Immunostaining and optical density of CD_31_ expression. **A**) Immunostaining for CD_31_ in the experimental groups. **B**) Effect of sorafenib (30 mg/kg), olmesartan (3, 10 or 30 mg/kg) and their combination on the optical density of CD_31_ immunostaining. **C**) Effect of concurrent administration of angiotensin (1–7) agonist (30 µg/kg/day, i.p.) or angiotensin (1–7) antagonist (3.3 mg/kg/trice/week, i.p.) and olmesartan on CD_31_ expression. Photomicrographs are captured at 200× magnification. EAC: Ehrlich's ascites carcinoma. Values are expressed as the mean ± S.E.M. and were analyzed using one-way ANOVA followed by Bonferroni's *post-hoc* test at *P*<0.05. *Significantly different from EAC-control. ^Δ^Significantly different from sorafenib monotherapy. •Significantly different from olmesartan (3 mg/kg) group. ^€^Significantly different from olmesartan (10 mg/kg) group.^$^Significantly different from olmesartan (30 mg/kg) group.^◊^Significantly different from the combination of olmesartan and angiotensin (1–7) agonist.

Immunostaining for intratumoral CD_31_ has been used to evaluate the degree of tumor angiogenesis as the density of the microvessels reflecting the degree of angiogenesis. The present results indicated that the EAC-control group showed the highest optical density for CD_31_among the study groups (Fig. 6 A and B). Monotherapy with olmesartan (3, 10 or 30 mg/kg) produced a significant, dose-dependent, reduction in microvessel density compared to the EAC-control group. Similarly, monotherapy with sorafenib produced a significant decrease in microvessel density compared to the EAC-control group. The combination of both olmesartan and sorafenib produced a marked reduction in the optical density of CD_31_compared to the EAC-control, as well as to the corresponding monotherapies (Fig. 6B). Moreover, concurrent administration of the angiotensin (1–7) agonist with olmesartan (30 mg/kg) reduced the microvessel density compared to the EAC-control group as well as to olmesartan (30 mg/kg) group (Fig. 6C). In contrast, concurrent administration of angiotensin (1–7) antagonist with olmesartan (30 mg/kg) increased in the optical density of microvessels compared to the monotherapy with olmesartan (30 mg/kg) (*P*<0.05, Fig. 6C).

## Discussion and Conclusion

Angiotensin II has been reported to promote tumor growth and angiogenesis [9]. Several tumor cell types, such as melanoma, pancreatic [28], renal [29], breast [30], bladder [31] and prostate cancers [32], have been reported to express the angiotensin II receptors. It has been previously reported that the high oxidative stress produced in EAC model causing chronic hypoxia and tissue injury [18], [33]. This chronic hypoxia increased the density of angiotensin II type 1receptors [34]. Therefore, EAC model can be considered as a good target for angiotensin II type 1 receptor blockers. Angiotensin II type 1 receptor blockade is reported to have inhibitory effects on many models of angiogenesis 34–37 and on the growth of microvessels induced by angiotensin II[38].Therefore, angiotensin II type 1 receptor blockers have been considered as an anti-angiogenic therapeutic option [39].

Angiotensin II receptor activation is associated with increased protein tyrosine phosphorylation and activation of the mitogen-activated protein kinases (MAPK), which leads to the activation of growth factors and cytokines [40]. Angiotensin II stimulates the phosphorylation of many non-receptor tyrosine kinases, including phospholipase C gamma, Src family kinases and Janus kinase receptors [40]. In addition, angiotensin II influences the activity of receptor tyrosine kinases, such as endothelial growth factor receptors, platelet-derived growth factor receptor and insulin-like growth factor receptor [41].

The current study was the first to measure the levels of IGF-I receptor in the EAC solid tumor model. Key et al. [42] noted that increased levels of IGF-I were associated with an increased cancer risk. In addition, the up-regulation of the IGF-I receptor and its cognate ligand IGF-I have been found in a variety of solid human tumors [43]. Our results agree with the previous results that the EAC-control group showed high serum levels of IGF-I and a greater expression of IGF-I receptor in the tumor cells. Furthermore, the angiogenic effect of IGF-I was evident as IGF-I was reported to induce tumor angiogenesis via the induction of VEGF [3]. In agreement, our results demonstrated that olmesartan (30 mg/kg) reduced the serum level of IGF-I, as well as VEGF. In addition, activation of the IGF-I receptor was reported to trigger the activation of the Ras–Raf MAPK and PI3K–protein kinase B pathways that converge with the VEGF production pathway [44].

In the current study, serum level of VEGF was high in the EAC-control group and this could be attributed to hypoxia induced in the tumor and the subsequent induction of the expression of VEGF and its receptor. Similarly, it has been reported that hypoxia inducible factor 1 alpha (HIF-1α) increased the expression of VEGF in experimental carcinogenesis [45]. Treatment with olmesartan (10 and 30 mg/kg) reduced the serum VEGF and down-regulated the expression of VEGF receptor-2 in the tumor discs. Similar to olmesartan, candesartan was reported to inhibit VEGF production and decrease prostate cancer growth [46]. Consistently, Rakusan et al. [34] reported that angiotensin II type 1 receptor-dependent angiogenic effects involve the activation of growth factors, such as VEGF, and inflammatory pathways. The combination treatment of olmesartan (30 mg/kg) and sorafenib significantly reduced the serum VEGF level compared to monotherapies, and this pointed to the potentiating effect of olmesartan on the anti-angiogenic effect of sorafenib.

Angiogenesis, the development of new blood vessels from pre-existing vasculature, is a prerequisite to tumor growth and its metastatic spread. Microvessel density reflects inter-capillary distance; it is influenced by both angiogenic and non-angiogenic factors. Microvessel density is considered to be a powerful candidate for prognosis [47] and many experimental studies used immunostaining for CD_31_ to determine microvessel density [33], [48]–[52]. In the current study, microvessel density, as indicated by CD_31_ immunostaining, the combination therapy produced a greater reduction in microvessel density compared to monotherapies; this indicates that the greater tumor inhibition exerted by the combination therapy was, at least in part, attributed to the combined anti-angiogenic effect of both drugs. Similar to the current results, microvessel density was reported to be reduced in prostate cancer cells treated with angiotensin II type I receptor blockers; this indicates that these drugs inhibit the vascularization of prostate cancer cells [46]. The loss of VEGF expression in a tumor results in a dramatic decrease in vascular density and vascular permeability [53].

The current data highlighted that concurrent administration of an angiotensin (1–7) receptor agonist along with olmesartan potentiated the antitumor activity of the latter. However, concurrent administration of the receptor blocker diminished the effect of olmesartan. In agreement, mice treated with angiotensin (1–7) showed reduced tumor cyclooxygenase 2 activity, suggesting that the heptapeptide may decrease the production of proinflammatory prostaglandins to inhibit tumor growth. Furthermore, the same study showed that angiotensin (1–7) inhibited the proliferation of lung cancer cells *in vitro* and reduced the serum-stimulated growth of three human lung adenocarcinoma cell lines [54]. The current results agree well with these findings; the combination of olmesartan (30 mg/kg) with the angiotensin (1–7) agonist reduced the serum level of VEGF and the expression of its receptors in the tumor discs, whereas addition of the angiotensin (1–7) antagonist (A-779 peptide) blocked this effect. These results indicated that the anti-angiogenic effect of olmesartan was, at least in part, dependent on angiotensin (1–7) receptors. Angiotensin (1–7) was reported to reduce tumor size by diminishing the blood supply to the tumor cells, thereby leading to tumor death [55].

The anti-tumor effect of olmesartan was confirmed in the present study where treatment with olmesartan reduced the tumor weight, assuming that this was linked to the angiostatic effect of olmesartan which resulted in tumor growth impairment. At the same time, the combinations of olmesartan (30 mg/kg) with the angiotensin (1–7) agonist reduced the tumor weight. However, the anti-tumor effect of olmesartan was reduced by the angiotensin (1–7) antagonist while the agonist potentiates it. These results suggest that the anti-angiogenic effect of olmesartan is mediated through the angiotensin (1–7) receptors. It was reported that sorafenib induced apoptosis in in cancer models process [56]–[58]. Similarly, angiotensin II type 1 receptor blockers induced apoptosis in experimental models [59]–[61]. Therefore, apoptosis may be a common mechanism underlying the anti-tumor effect of sorafenib and olmesartan in the treatment of cancer.

In conclusion, the present results showed that olmesartan (30 mg/kg) potentiated the anti-angiogenic effect of sorafenib- through inhibition of IGF-I, VEGF and their receptors- leading to greater anti-tumor activity. The anti-angiogenic effect of olmesartan was, at least in part, mediated through the angiotensin (1-7) receptor. Therefore, the present study highlighted the beneficial role of olmesartan as an adjuvant medication to sorafenib in the treatment of cancer. This combination could be of choice as sorafenib was reported to increase blood pressure in cancer patients [16] and prescribing an antihypertensive remedy might be essential in some patients.
